# Treatment of Atopic Dermatitis Using a Full-Body Blue Light Device (AD-Blue): Protocol of a Randomized Controlled Trial

**DOI:** 10.2196/11911

**Published:** 2019-01-08

**Authors:** Christian Kromer, Viktoria P Nühnen, Wolfgang Pfützner, Sebastian Pfeiffer, Hans-Joachim Laubach, Wolf-Henning Boehncke, Joerg Liebmann, Matthias Born, Michael P Schön, Timo Buhl

**Affiliations:** 1 Department of Dermatology, Venereology, and Allergology University Medical Centre Göttingen Göttingen Germany; 2 Clinic for Dermatology and Allergology University of Marburg Marburg Germany; 3 Clinical Trials Unit University Medical Centre Göttingen Göttingen Germany; 4 Division of Dermatology and Venereology Geneva University Hospital Geneva Switzerland; 5 Department of Pathology and Immunology University of Geneva Geneva Switzerland; 6 Philips GmbH Innovative Technologies Aachen Germany; 7 Lower Saxony Institute of Occupational Dermatology University Medical Centre Göttingen Göttingen Germany

**Keywords:** atopic dermatitis, atopic eczema, blue light, irradiation, ultraviolet light

## Abstract

**Background:**

Irradiation with visible blue light (wavelength 400-495 nm) is a promising, effective, and safe new treatment option for chronic inflammatory skin diseases such as psoriasis and atopic dermatitis.

**Objective:**

We will perform a multicenter, placebo-controlled, double-blinded, 3-armed, prospective, randomized controlled trial to investigate the efficacy and safety of full-body blue light devices (wavelengths: 415 nm and 450 nm) compared with that of placebo irradiation for the treatment of atopic dermatitis.

**Methods:**

We are planning to enroll a total of 150 patients at the University hospitals in Göttingen (Germany), Marburg (Germany), and Geneva (Switzerland).

**Results:**

The trial was approved by the lead ethics committee of the medical faculty of the University of Göttingen (21/11/16). Further approvals were obtained from local and federal authorities (ethics committee Marburg, Cantonal Commission for Research Ethics Geneva, Suisse Medic, and Bundesinstitut für Arzneimittel und Medizinprodukte).

**Conclusions:**

We will disseminate the results in a peer-reviewed journal.

**Trial Registration:**

ClinicalTrials.gov NCT03085303; https://clinicaltrials.gov/ct2/show/NCT03085303 (Archived by WebCite at http://www.webcitation.org/73ucqkkA1)

**International Registered Report Identifier (IRRID):**

DERR1-10.2196/11911

## Introduction

### Background

Atopic dermatitis is a common chronic inflammatory disease of the skin with a lifetime prevalence of 10%-20% in adults in developed countries [1]. Mutations in the filaggrin gene were shown to play a key role in the etiology of the disease [2]. Patients present with itchy, red, dry or oozing lesions primarily at flexural body areas. Disease severity can be measured using the Eczema Area and Severity Index (EASI), which assesses redness, thickness, scratching, and lichenification on a scale of 0 (none) to 3 (severe) for different body regions [3]. SCORing Atopic Dermatitis (SCORAD) and Patient Oriented-SCORing Atopic Dermatitis (PO-SCORAD) are instruments that additionally consider the subjective symptoms of pruritus and insomnia [4,5]. The Investigator’s Global Assessment (IGA) is a score to assess overall disease severity on a scale of 0 (clear) to 4 (severe) [6]. Disease-related impairment of quality of life can be measured using the Dermatology Life Quality Index (DLQI) [7].

As atopic dermatitis is a chronic disease with an unpredictable and often relapsing course, long-term control is necessary [8]. In clinical practice, a stepwise approach is used, starting with active agent-free lipid balancing creams, cortisone-containing topical agents, and conventional ultraviolet (UV) irradiation for mild to moderate disease forms. Immunosuppressive medication is employed in more severely affected patients. Cortisone- containing topical agents effectively reduce inflammation but may also cause infection and skin atrophy in long-term use [9]. Conventional UV irradiation bears the risk of developing skin cancer and promotes accelerated skin aging [8]. Recently, a new treatment modality, UV-free blue light irradiation (wavelengths between 400 and 495 nm), was found to effectively improve inflammatory skin diseases at a favorable risk profile [10-13].

### Preliminary Data With Blue Light

Clinical data on blue light in chronic inflammatory skin diseases are scarce. Psoriasis and eczema have been the exclusive indications for comprehensive clinical trials in the past. Regarding eczema, Keemss et al [11] reported on 21 patients with mild to moderate disease who were locally treated with blue light-emitting diode (LED) light (wavelength: 453 nm) for 30 minutes 3 times per week for 4 weeks. No adverse events (AEs) occurred. The local Eczema Severity Index, which rates erythema, infiltration, lichenification, and crusts on a scale of 0 to 3, decreased on average by 1.9 (SD 2.02) in treated areas compared with that by 1.3 (SD 2.24) in untreated areas. Becker et al [10] investigated the treatment of 36 patients with severe atopic dermatitis with a full-body blue (FBB) light irradiation device (wavelengths between 400 and 500 nm accounted for 66% of the emission spectrum of the light source). Treatment consisted of 1 cycle of consecutive, daily blue light irradiations over 5 days. Each side of the body was irradiated for 24 minutes. Cycles were repeated in case of disease exacerbation, triggered by the patient’s demand. The EASI score in the treated patients improved by 41% and 54% after 3 and 6 months, respectively. The following transitory mild AEs were reported: local redness, warmth, and itching of the skin.

Other smaller studies investigated the effect of blue light on patients with psoriasis. Pfaff et al [12] used a localized blue light device (wavelength: 453 nm) to treat 49 patients with mild psoriasis for 30 minutes daily or almost daily for 12 weeks with 4 weeks of follow-up. After 78-90 days, the Local Psoriasis Severity Index significantly decreased compared to an untreated area (mean 0.92 [SD 1.10]). No treatment-related AEs occurred. Weinstabl et al [13] reported on localized irradiation of 40 patients with mild to moderate psoriasis with blue light. Group 1 (n=20) received treatment at home with blue light (LED, emission maximum: 420 nm) for 15 minutes once daily for 4 weeks (2 weeks follow-up). Group 2 (n=20) received treatment with another blue light device (LED emission maximum: 453 nm). The contralateral control plaques remained untreated in both groups. In both groups, the Local Psoriasis Severity Index significantly decreased in treated areas compared with that in untreated areas. No severe AEs occurred. Slight transient hyperpigmentation was noted after 3 weeks in both groups (59% and 50%, respectively). However, an FBB light device has not been clinically tested outside the mentioned studies.

## Methods

### Study Design

AD-Blue trial is a multicenter, placebo-controlled, double- blinded, 3-armed, prospective, randomized controlled trial (European Database on Medical Devices #CIV-16-11-017565 and ClinicalTrials.gov NCT03085303). We designed the clinical study following the Standard Protocol Items: Recommendations for Interventional Trials guidelines (see Multimedia Appendix 1).

### Objectives

The primary objective is to estimate the efficacy of irradiation with blue light (415 nm and 450 nm) compared with placebo irradiation in adult patients with atopic dermatitis, determined using multiple clinical scores, such as EASI and SCORAD. Secondary objectives comprise the assessment of safety, tolerability, and satisfaction with irradiation with blue light as well as the time until treatment response under therapy and the duration of response after the last irradiation.

### Endpoints

The primary endpoint is to determine the change from baseline in EASI in a comparison between treatment arms and placebo arm at the end of treatment. Secondary endpoints include the change from baseline in SCORAD, PO-SCORAD, visual analog scale of itch, DLQI, and IGA scores; the proportion of patients achieving 50% reduction from baseline EASI score (response); the so far mentioned endpoints at follow-up; and time until treatment response. The following safety endpoints will be addressed: change from baseline in hyperpigmentation of treated skin exposed to blue light and control as well as AEs (serious and nonserious), adverse device events (serious and nonserious), and device deficiencies.

### Study Population

A total of 150 patients with atopic dermatitis will be recruited at 3 sites: Geneva, Marburg, and Göttingen. The recruitment will be independent of the severity of disease and is limited to 50.0% (75/150) at each site. A sample size of 50 patients per group will have 84% power to detect a mean effect size of 0.6.

### Recruitment and Status of the Study

The first enrollment took place in March 2017. The study duration per patient is 13 weeks (1 week of screening, 8 weeks of irradiation, and 4 weeks of follow-up), and the estimated total time frame for recruitment of 150 patients is 14 months (last visit of the last patient estimated to be in August 2018). During July to September, no irradiations will take place due to the possible improvement of disease severity linked to the increased natural sun exposure.

### Inclusion and Exclusion Criteria

Patients aged 18-75 years with atopic dermatitis according to the UK criteria of atopic dermatitis [14] and a body mass index between 18 and 35 kg/m^2^ who are willing to abstain from excessive sun or UV exposure and have given written informed consent will be eligible. Patients with a severe past or present disease that may affect the outcome of the study, including HIV or hepatitis B or C; patients with a risk of noncompliance with study procedures; pregnant or nursing women; patients with alcohol or drug abuse within 12 months prior to screening; patients with photodermatosis, photosensitivity, immunodeficiency, or genetic deficiencies with increased sensitivity to light; and patients who have been diagnosed with invasive skin cancer at any time or with severe actinic damage present at baseline visit will be excluded from the trial.

We will prohibit the following concomitant medication within a certain timeframe before and during the study: systemic immunosuppression treatment such as glucocorticoids, cyclosporine, azathioprine, and mycophenolate mofetil (within 8 weeks prior to baseline visit); UV irradiation treatment (within 4 weeks prior to baseline visit); topical steroids or calcineurin inhibitors (within 2 weeks prior to baseline visit); and photosensitizing medication and colors on the patient’s skin (within 3 days prior to baseline visit). Active agent-free lipid balancing creams (Unguentum leniens) will be allowed during the trial and handed out on request.

### Investigational Devices

**Figure 1:**
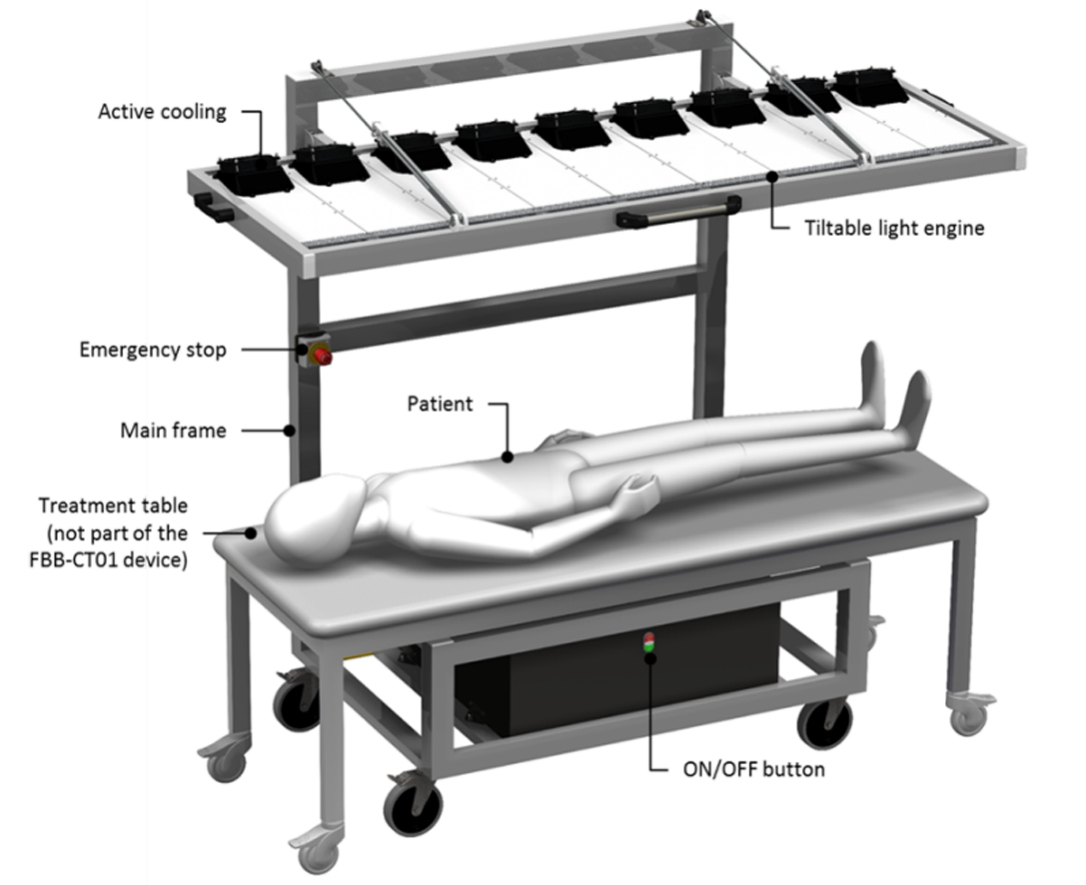
Design of the FBB-CT01 device (415 nm and 450 nm). The patient lies on the treatment table and receives treatment from the light engine above.

We will investigate FBB-CT01 devices (Philips; Aachen, Germany; not Conformité Européene, CE, marked) with LEDs emitting blue light (Figure 1). This will be done by using the following three different parameter settings:

1. 415 nm device: Device equipped with 415 nm wavelength LEDs. Light output=40 mW/cm^2^. Light module equipped with fans.
2. 450 nm device: Device equipped with 450 nm wavelength LEDs. Light output=40 mW/cm^2^. Light module equipped with fans.
3. Placebo device: Device equipped with 450 nm wavelength LEDs. Light output=0.2 mW/cm^2^. Light module without fans.

The devices are on lock wheels for easy transportation. We will administer phototherapeutic light for 15 minutes to each side of the body of the patient (30 minutes in total for both sides) who will lie flat on a treatment table. We will provide specific protective goggles to filter the blue color of the light. Once the user presses the ON button to switch on the device, all treatment LEDs will be on. If the user needs to stop the treatment, he or she will either press the OFF button or, in case of an emergency, the “emergency stop” button.

### Methodology

We will randomize 150 patients diagnosed with atopic dermatitis 1:1:1 to arm 1 (irradiation for 30 minutes at 415 nm wavelength), arm 2 (irradiation for 30 minutes at 450 nm wavelength), and arm 3 (irradiation for 30 minutes at low-dose, placebo). We will stratify randomization by study site and initial EASI score (assessed during screening visit) with permuted and variable block sizes. For each strata combination, a list of separate randomization sequences will be generated using SAS 9.3 (Cary, NC). Irradiation will be scheduled 3 times a week for 8 weeks. Participants will be followed up for 4 weeks after the last irradiation. Patients will be blinded by wearing tinted protective goggles that disenable the distinction of different wavelengths of light. Medical doctors will examine patients in rooms other than those equipped with the investigational medical devices. These examiners are blinded and are, therefore, not involved in the preparation and process of irradiation. If the safety of an enrolled patient is potentially jeopardized, the investigator can break the blinding of the treatment. According to the predefined study schedule, we will measure skin pigmentation with a mexameter; take photos and blood samples; measure blood pressure; and gather data on Fitzpatrick skin type, DLQI, itch on visual analog scale, SCORAD, IGA, EASI, and PO-SCORAD scores. Detailed information on assessments is depicted in the study schedule (Multimedia Appendix 2). If the EASI score increases by 50% or more compared to baseline after at least 4 weeks of treatment (earliest: day 28=visit 13), a rescue scheme can be applied that allows the use of topical steroids or antihistamines. The investigator will decide whether to prescribe these medications, in what doses, and for how long. The respective patient will not drop out, but the rescue medication as well as the day of rescue will be documented. The study database will be provided by the Universitätsmedizin Göttingen, Ressort Forschung und Lehre, Referat Klinisches Studien Management using secuTrial, which is a browser-based electronic data capture system compliant with Good Clinical Practice (audit trail, role-rights concept). The database will be accessible via the internet.

### Patient and Public Involvement

No patients or public were involved in the design of this study.

### Statistical Considerations

The primary analysis will be performed based on the full analysis set following the intention-to-treat principle. Dropouts during first 4 weeks of treatment will be replaced. Patients missing more than 5 irradiation appointments in total until day 25 (inclusive) or more than 3 consecutive irradiation appointments until day 25 (inclusive) will be considered dropouts and will be replaced. Dropouts after day 25 of the study protocol will not be replaced and will be analyzed in the intention-to-treat population. Sensitivity analysis will be done on the per-protocol population. The difference between the end of treatment after 8 weeks and baseline in primary effectiveness endpoint EASI will be analyzed using pairwise 2-sample *t* tests between each of both active treatments against placebo. As sensitivity analysis, an analysis of covariance will be used with study site, gender, and the hand out of topical emollient as additional factors and the EASI baseline value, age, and Fitzpatrick skin type as covariates. Other continuous secondary endpoints will be analyzed analogously. The EASI response will be analyzed primarily using chi-square test and as a sensitivity analysis with logistic regression. Time until treatment response will be analyzed using Kaplan-Meier estimator and log-rank test. In a further sensitivity analysis, if both wavelengths show better effectiveness than placebo, a pooled comparison against placebo will be done to increase the sample size.

The two comparisons, namely, 415 nm versus placebo and 450 nm versus placebo will be evaluated equally. To address the multiplicity, a success will be declared if both comparisons are statistically significant at an alpha level of .05 or if one of these two is statistically significant with respect to half of alpha at .025.

The two risk differences between one of each blue light irradiation treatments against placebo of the rates of patients with at least one device-related adverse event (rated as possible, probable, or certain) will be compared using asymptotic Wald test of noninferiority for the risk difference. Missing values for the primary and secondary endpoints will be replaced using proper single regression methods (IVEware; University of Michigan, Ann Arbor, MI). Besides the scores (such as EASI scores), the study site, study visits with the documented EASI score, treatment group, gender, age, hand out of topical emollient, and Fitzpatrick skin type will be included in the regression models. In safety analysis, per-protocol analysis, and explorative analysis, missing values will not be replaced.

After the completion of the treatment phase of about 75 patients, first, the EASI score difference between the end of treatment and baseline and, second, the rates of device-related AEs will be analyzed descriptively using *t* test and Fisher’s exact test, as appropriate (interim analysis). Based on the estimates, it will be evaluated whether a total of 150 patients will be sufficient to show superiority against placebo at the end of the study.

### Ethics and Dissemination

This investigation will be performed according to the Declaration of Helsinki [15], International Organization for Standardization (ISO) 14155, European Medical Device Vigilance System (MEDDEV) 2.3/7 serious adverse event reporting, and other applicable regional and national regulations (eg, for Europe, the Medical Device Directive, Active Implant Medical Device Directive, MEDDEV, and other ISO norms for specific medical devices). The sponsor has taken out an insurance policy for the total duration of the study. This insurance policy covers the patients in respect of the risks involved in this study. Before initiation of the study, the protocol, the patient information sheet, and the consent form were presented to and approved by the independent ethics committee of the medical faculty of the University of Göttingen (lead ethics committee; 21/11/16). Further approvals were obtained from local and federal authorities (EC Marburg, Cantonal Commission for Research Ethics Geneva, Suisse Medic, and Bundesinstitut für Arzneimittel und Medizinprodukte [BfArM]). The names of patients and all confidential data are subject to professional discretion and the Bundesdatenschutzgesetz. Processing of medical data will only take place in pseudonymous form.

During the screening visit and preceding any study-mandated procedure, the patient will receive a copy of the written patient information sheet containing a complete and comprehensive explanation of the significance, nature, extent, and possible risks of the study and the statement that the patient is free to withdraw from the study at any time without any negative consequences. In addition, a physician will conduct an oral information session during which the patient will be given sufficient time and opportunity to clarify any questions. After this, a written informed consent will be given to the patients for a dated signature. After signature, the patient will receive a copy of the signed consent form. In addition, the patients will be asked to grant consent for taking blood and skin samples for additional investigations outside the current clinical trial. This is not mandatory and does not affect clinical trial participation in general.

### Safety and Risk to Benefit Rationale

Foreseeable AEs include thermal discomfort during the irradiation and increased skin pigmentation, which we expect to be of a very mild nature as experienced in previous trials. Due to the absence of UV light in our devices, we do not expect dermatitis solaris (sunburn) or an increased risk for melanoma or nonmelanoma skin cancer.

Unexpected physical accidents during the irradiation procedure itself (eg, the patient falling off the examination bed or a deficient device harming participants’ physical integrity) are possible out of general considerations. The non-CE mark investigational medical devices are classified as Class IIa according to Annex IX of the Directive 93/42/EEC and as Risk Group 2 devices according to EN 60601-2-57. They have been approved by competent federal authorities (BfArM, Suisse Medic). Precautions have been taken by means of risk management, testing, and protocol design to protect the health and safety of the patients and personnel participating in the clinical study. All AEs will be recorded and documented. Serious AEs will be reported in accordance with the appropriate legal regulations (Medizinprodukte-Sicherheitsplanverordnung). Due to the well-documented efficacy of blue light treatment in psoriasis and eczema, clinical benefits include an improvement in objective disease severity and quality of life (less itching or less insomnia expected).

In summary, blue light has the potential to improve inflammatory skin diseases. Associated risks are rare, mild, and transitory. After re-evaluation of the risks, the overall residual risk has been concluded to be satisfactory. As an overall result, we conclude that the benefits for the patients outweigh residual risk, thereby justifying the introduction of the device.

## Results

The project was funded by Philips Light & Health, and enrollment was completed in May 2018. Data analysis is currently underway, and the first results are expected to be submitted for publication in January 2019.

## Discussion

This is the first international, multicenter, randomized controlled trial investigating the effect of treatment with FBB light devices in patients with atopic dermatitis. A broad range of validated outcome measures has been applied to account for objective and subjective symptoms. This is an investigator-initiated trial with the design developed at the academic institutions. However, clinical heterogeneity and placebo effects may dilute the intervention’s effects.

## Appendix

Multimedia Appendix 1 SPIRIT Checklist.

Multimedia Appendix 2 Study schedule of assessments.

## Abbreviations

AE: adverse event
BfArM: Bundesinstitut für Arzneimittel und Medizinprodukte
DLQI: Dermatology Life Quality Index
EASI: Eczema Area and Severity Index
FBB: full-body blue
IGA: Investigator’s Global Assessment
ISO: International Organization for Standardization
LED: light-emitting diode
MEDDEV: European Medical Device Vigilance System
PO-SCORAD: Patient-Oriented-SCORing Atopic Dermatitis
SCORAD: SCORing Atopic Dermatitis
UV: ultraviolet
