# Supplementary material for: Treatment of Atopic Dermatitis Using a Full-Body Blue Light Device (AD-Blue): Protocol of a Randomized Controlled Trial
Source: JMIR Res Protoc. 2019 Jan 8;8(1):e11911. doi: 10.2196/11911 (PMC6329412; doi:10.2196/11911)
Supplement: Multimedia Appendix 2 [file resprot_v8i1e11911_app2.pdf]

Multimedia Appendix 2. Study schedule of assessments.

		Treatment																								
**Visit No.**	**Screening**	1	2	3	4	5	6	7	8	9	10	11	12	13	14	15	16	17	18	19	20	21	22	23	24	**Follow-up** (25)
**Week**		1			2			3			4			5			6			7			8			12
Irradiation		X	X	X	X	X	X	X	X	X	X	X	X	X	X	X	X	X	X	X	X	X	X	X	X	
DLQI [a]		X						X						X						X					X	X
Photos		X																							X	X
Itch VAS[b]		X						X			X			X			X			X					X	X
SCORAD [c]		X						X						X						X					X	X
IGA [d]		X						X						X						X					X	X
EASI [e]	X	X						X						X						X					X	X
PO-SCORAD [f]		X						X			X			X			X			X					X	X
Adverse events		X	X	X	X	X	X	X	X	X	X	X	X	X	X	X	X	X	X	X	X	X	X	X	X	X
Blood pressure	X	X						X						X						X					X	X
Fitzpatrick skin type		X																								
differential blood count	X	X						X						X						X					X	X
IgE, sx1, HIV, Hep B/C, preg test (serum)	X																									
Patient- and disease-related info		X																							X	
Mexameter pigmentation		X												X											X	

Abbreviations: [a] Dermatology Life Quality Index, [b] Visual Analogue Scale, [c] SCORing Atopic Dermatitis, [d] Investigator Global Assessment, [e] Eczema Area and Severity Index, [f] Patient oriented SCORing Atopic Dermatitis. Procedures with green background will be performed by the blinded investigator, procedures in red will be performed by the study nurse.
